# Cerebral venous thrombosis with hyperhomocysteinemia due to loss of heterozygosity at methylenetetrahydrofolate reductase (*MTHFR*) locus: a case report

**DOI:** 10.1186/s12883-023-03200-y

**Published:** 2023-04-19

**Authors:** Mingjie Zhang, Bingxin Shi, Mangsuo Zhao

**Affiliations:** 1 Department of Neurology, The First Medical Center, Chinese PLA General Hospital, No. 28 Fuxing Road, Beijing, 100853 PR China; 2 Brain Science Center, Tsinghua University Yuquan Hospital, No. 5 Shijingshan Road, Beijing, 100049 PR China

**Keywords:** Cerebral venous thrombosis, Hyperhomocysteinemia, Methylenetetrahydrofolate reductase, Loss of heterozygosity

## Abstract

**Background:**

Loss of heterozygosity (LOH) at methylenetetrahydrofolate reductase (*MTHFR*) locus has been reported in tumor tissue. But the mutation was never reported in cerebral venous thrombosis (CVT) with hyperhomocysteinemia (HHcy) before.

**Case presentation:**

A 14-year-old girl was admitted with an intermittent headache and nausea for 2 months. The plasma homocysteine level was 77.2 µmol/L. Lumbar puncture revealed an intracranial pressure > 330 mmH2O. Cerebral MRI and MRV revealed superior sagittal sinus thrombosis. Whole-exome sequencing revealed LOH at Chr1:11836597–11,867,232 affects exons 10–21 of *C1orf167*, the entire *MTHFR*, and exons 1–2 of the *CLCN6* gene. The normal allele was the c.665 C > T/677 C > T variant in *MTHFR*. The patient was treated with nadroparin for 2 weeks, followed by oral rivaroxaban. Supplemental folate and vitamins B12 and B6 were prescribed. One month later, she had no headache and the intracranial pressure had decreased to 215 mmH2O. MRI showed shrinkage of the thrombosis in the superior sagittal sinus, the degree of stenosis had significantly decreased.

**Conclusions:**

Rare LOH at the MTHFR locus should be analyzed in CVT with HHcy. With anticoagulation treatment, the prognosis was good.

## Background

Cerebral venous thrombosis (CVT) is a rare cerebrovascular disease that affects about five people per million and accounts for 0.5% of all strokes [1]. Several disorders can cause or predispose patients to CVT. Hyperhomocysteinemia (HHcy) is a strong, independent risk factor for CVT and is present in 27–43% of patients and 8–10% of controls, with an odds ratio of 4–7 [2–4]. Causes of HHcy include genetic mutations and deficiencies of the enzymes 5,10-methylenetetrahydrofolate reductase (MTHFR), methionine synthase, and cystathionine β-synthase. HHcy can also be caused by deficiencies in vitamins B6 (pyridoxine), B9 (folate), and B12 (cobalamin), which influence methionine metabolism. Furthermore, HHcy can be caused by a rich diet and renal impairment [5]. More than 100 *MTHFR* mutations have been described as causing severe MTHFR deficiency [6]. Loss of heterozygosity (LOH) at the MTHFR locus has been described in tumor tissue through polymerase chain reaction-restricted fragment length polymorphism (PCR-RFLP) technique and microsatellite analyses [7–10]. Loss of the variant ‘T’ allele was observed with preservation of the wild-type allele in most of the cancer tissue, resulting in a ‘C’ hemizygote state [10]. This paper is the first to report a case of superior sagittal sinus thrombosis with HHcy due to LOH at the *MTHFR* locus.

## Case presentation

A 14-year-old girl was admitted with an intermittent headache and nausea for 2 months. Her mother and aunt suffered from migraine. Her grandmother died of amyotrophic lateral sclerosis. Two months earlier, the patient developed a mild headache that worsened gradually over the next 3 days. The pain was located on the forehead and described as throbbing and swelling, with a visual analogue scale score of 9/10. It was accompanied by nausea, vomiting, and extreme sensitivity to light and sound. No other symptoms emerged, such as fever, hearing loss, dysphagia, hemiplegia, or hemianesthesia. Cranial computed tomography (CT) in the emergency room was normal. An ophthalmologist found a normal intraocular pressure and fundus. Migraine was suspected and oral ibuprofen and acupuncture were prescribed. The headache disappeared completely after 1 week. However, a similar headache and nausea developed 2 months later. The headache was persistent, but relieved for several hours after oral ibuprofen.

In examinations, no cognitive disturbances were noted. Bilateral abducens nerve palsy was noted, but without diplopia. Strength in the upper and lower extremities was 5/5. The finger-to-nose and heel-knee-tibia tests were normal. Romberg sign was negative. Tendon reflexes in the extremities were normal. The plantar responses were down-going bilaterally. The neck was stiff and the chin–sternum straight distance was 3 cm. Both the Kernig and Brudzinski signs were negative.

**Fig. 1:**
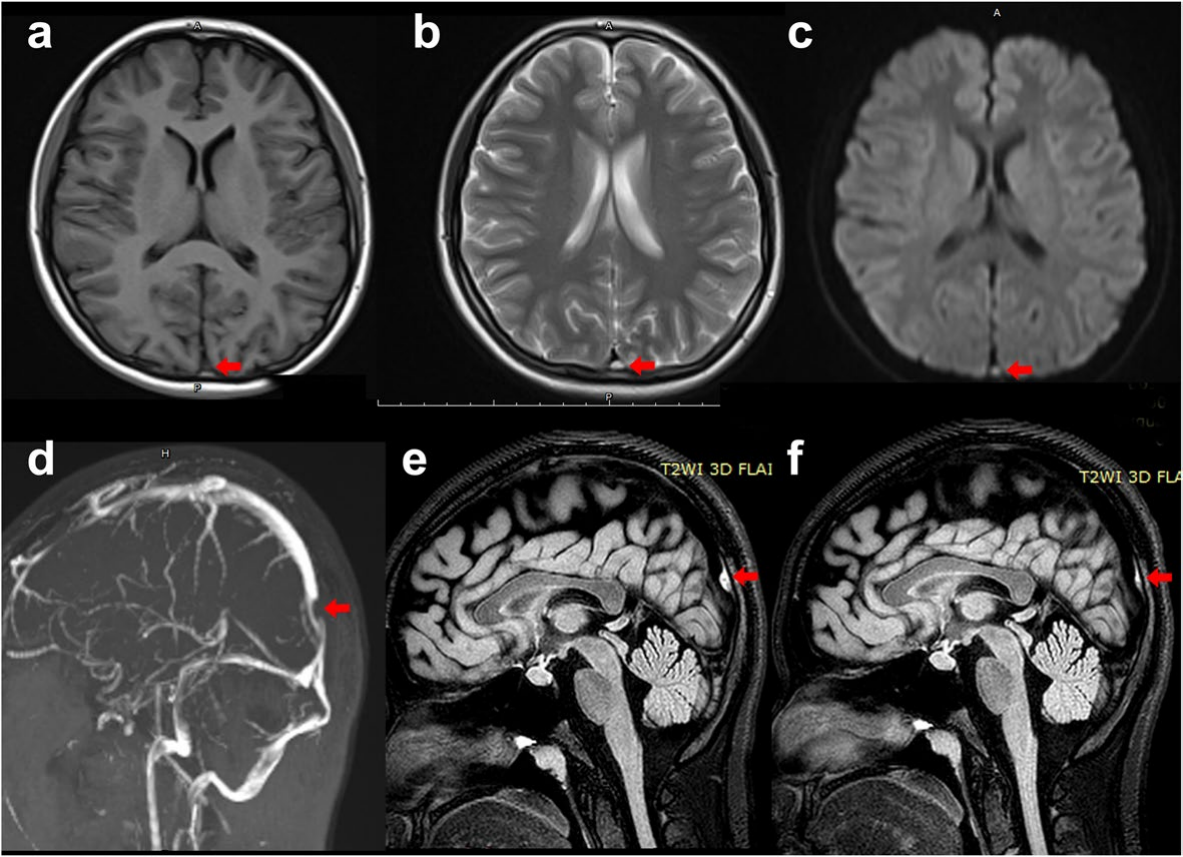
Cerebral MRI and MRV of the patient Magnetic resonance imaging (MRI) showed abnormal signal lesions in the superior sagittal sinus, which was isointense on TW1I (**a**) and hyperintense on TW2I (**b**) and diffusion-weighted images (**c**). Magnetic resonance venography (MRV) strongly suggested stenosis of superior sagittal sinus (**d**) MRI showed stenosis of superior sagittal sinus and abnormal signal lesions in the superior sagittal sinus, which was hyperintense in fluid attenuated inversion recovery (FLAIR) after admission (**e**). After one month, MRI showed shrinkage of the thrombosis in the superior sagittal sinus; the degree of stenosis had significantly improved (**f**)

The laboratory work-up was normal, including routine blood tests, urinalysis, stool analyses, liver, renal, and thyroid function, erythrocyte sedimentation rate, C-reactive protein. High-density lipoprotein was 0.83 (normal 0.94–2.5) mmol/L; low-density lipoprotein and triglyceride levels were normal. The prothrombin time was 13.7 s (normal 9.4–12.5) and activated partial thromboplastin time and d-dimer levels were normal. The plasma homocysteine (Hcy) level was 77.2 (normal < 15) µmol/L. Folate concentration was 5.15 (normal 9.53–44.9) nmol/L and vitamin B12 levels were normal. The prolactin level was 670.2 (normal 102–496) mIU/L. There was no serum anti-nuclear, anti-double-stranded DNA, extractable nuclear antigen, anti-neutrophil cytoplasmic, or anti-cardiolipin antibodies or rheumatoid factor. Lumbar puncture performed in the supine position after admission revealed an intracranial pressure > 330 mmH2O, normal cerebrospinal fluid cell counts and protein levels, and normal microbiology results. Chest CT, electrocardiogram, and video-electroencephalogram results were normal. Ultrasound showed no disorders of the liver, gallbladder, spleen, pancreas, or kidneys. Peripheral vascular ultrasound indicated that the veins of both upper and lower limbs and the iliac vein were normal. Magnetic resonance imaging (MRI) showed abnormal signal lesions in the superior sagittal sinus, which was isointense to gray matter on TW1I, hyperintense to gray matter on TW2I and diffusion-weighted images (Fig. 1a, b, c). Magnetic resonance venography (MRV) strongly suggested superior sagittal sinus stenosis (Fig. 1d). Susceptibility-weighted imaging showed no abnormal signals.

The patient was treated with 4100 IU nadroparin for 2 weeks, followed by oral rivaroxaban 15 mg bid. Intravenous mannitol was prescribed to lower the intracranial pressure. Supplemental folate and vitamins B12 and B6 were prescribed. After 3 days, the headache was obviously relived. One week later, PT and prolactin levels had returned to normal. In repeat lumber puncture, the intracranial pressure had decreased to 270 mmH2O. Two weeks later, the headache had disappeared completely. One month later, she had no headache and the intracranial pressure had decreased to 215 mmH2O. Hcy levels had decreased to 27.9 µmol/L, while the folic acid level had increased to more than 45.4 nmol/L. MRI showed shrinkage of the thrombosis in the superior sagittal sinus; the degree of stenosis had significantly decreasedon fluid attenuated inversion recovery (FLAIR) (Fig 1 e, f). After 3 months, the oral rivaroxaban was stopped and she remained stable for the following two years.

## Genetic analyses

**Fig. 2:**
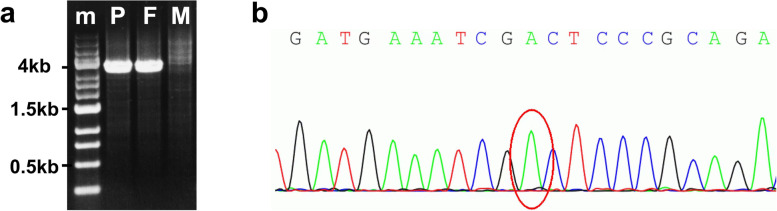
Breakpoint analysis of loss of heterozygosity The primers were designed for polymerase chain reaction (PCR) from Chr 1: 11834885 to Chr 1: 11868856. Because of the distance between the primer-binding sites on the normal allele (34 kb), only amplification of the deletion-carrying allele was possible and resulted in a 4 kb amplicon (deletion from Chr 1: 11836597 to Chr 1: 11867232). PCR showed 4 kb amplicon in the proband and her father (**a**). The normal allele was the c.665 C > T/677 C > T (NM_005957.4: c.665 C > T/p. Ala222Val, rs1801133:C > T) variant in *MTHFR* (**b**). *m* Marker, *P* Proband, *F* Father, *M* Mother

Whole-exome sequencing revealed LOH at Chr1:11836597–11867232 affects exons 10–21 of C1orf167, the entire *MTHFR*, and exons 1–2 of the *CLCN6* gene. The normal allele was the c.665 C > T/677 C > T (NM_005957.4: c.665 C > T/p. Ala222Val, rs1801133:C > T) variant in *MTHFR* (Fig. 2b), resulting in a ‘T’ hemizygote state. To analyze the breakpoint, primers MTHFR-F (5’ACTGGGTTACTGAATAGGAAATGACT-3’) and MTHFR-R (5’CAGTCCCATCAGAGTAGCATATTAAA-3’) were designed for polymerase chain reaction (PCR) from Chr 1: 11834885 to Chr 1: 11868856. Because of the distance between the primer-binding sites on the normal allele (from Chr 1: 11834885 to Chr 1: 11868856: 34 kb), only amplification of the deletion-carrying allele was possible and resulted in a 4 kb amplicon (deletion from Chr 1: 11836597 to Chr 1: 11867232). Deletion of Chr1:11836597–11,867,232 was confirmed in the proband and her father was a carrier of the mutation (Fig. 2a). Her mother had the homozygous c.665 C > T variant in *MTHFR*.

## Discussion and conclusions

A clinical presentation with a 2-month history of headaches and nausea is a classic sign of CVT. In the present case, high intracranial pressure was found via lumber puncture. The diagnosis of superior sagittal sinus thrombosis was ultimately established via MRI and MRV. Anticoagulant therapy was effective, supporting the diagnosis of CVT. Typical MRI finding of dural sinus thrombosis is showed isointense on T1WI, hyperintense on T2WI and hyperintense on DWI which is different from giant arachnoid granule which is hypointense on T1WI and DWI, hyperintense on T2WI and isointense on FLAIR [11, 12]. Follow-up MRI showed shrinkage of the superior sagittal sinus thrombosis, which also ruled out a diagnosis of a giant arachnoid granule.

The diagnostic work-up discovered HHcy, which is a known independent risk factor for CVT. The mechanisms by which HHcy cause thrombosis are still under investigation, but may include its toxicity toward endothelial cells, the promotion of smooth muscle cell proliferation and intimal thickening, decreased generation of nitric oxide and prostacyclin, and increased platelet adhesion and activation of Factor V [3]. The causes of HHcy include genetic mutations and enzyme deficiencies in MTHFR. In this case, the LOH affected exons 10–21 of C1orf167 (an uncharacterized protein) and exons 1–2 of *CLCN6* (a voltage-gated chloride channel). Neither has been reported to be related to HHcy or vascular disorders. The normal allele was c.665 C > T/677 C > T (NM_005957.4: c.665 C > T/p. Ala222Val, rs1801133:C > T), while the variant in MTHFR resulted in a ‘T’ hemizygote state. Based on existing data on MTHFR activity, a ‘T’ hemizygote would have only 15% of the normal MTHFR activity, which is lower than the 30% normal MTHFR activity in TT homozygote cells [13]. The mutation caused high plasma levels of homocysteine in this case. Her father carried the mutation, but had no history of occlusive artery disease or venous thrombosis.

*MTHFR* C677T polymorphisms are major factors influencing folate status. Individuals with the TT genotype have lower serum folate concentrations and higher serum Hcy concentrations than those with the CC genotype [14]. Nishio K et al. suggested that individuals with the TT genotype may need to consume more folate (approximately 1.4 times more) to maintain serum folate levels similar to those found in individuals with the 677CC/CT genotypes [15]. The patient in the paper had a well-balanced diet, but the serum folate concentration decreased. After oral folate supplements for about 1 month, the patient’s serum levels increased to above the normal range; the plasma level of homocysteine decreased but HHcy remained constant. It shows that HHcy would not be reversed completely by oral folate supplements in patients with ‘T’ hemizygote mutation in *MTHFR*. No independent association has been identified between the c.665 C > T mutation in *MTHFR* and CVT. So far it is unknown if ‘T’ hemizygote mutation in MTHFR is associated with CVT independently, which need further research.

HHcy is a strong, independent risk factor for CVT and the plasma level of homocysteine should be part of the diagnostic work-up for CVT. We reported a case of cerebral venous sinus thrombosis with HHcy due to LOH at *MTHFR* locus for the first time. Rare LOH at the MTHFR locus should be analyzed in CVT with HHcy. With anticoagulation treatment, the prognosis was good.

## Data Availability

Not applicable.

## Abbreviations

LOH: Loss of heterozygosity
MTHFR: Methylenetetrahydrofolate reductase
CVT: Cerebral venous thrombosis
HHcy: Hyperhomocysteinemia
PCR-RFLP: Polymerase chain reaction-restricted fragment length polymorphism
MRI: Magnetic resonance imaging
MRV: Magnetic resonance venography
FLAIR: Fluid attenuated inversion recovery.
